# Pyoderma gangrenosum diagnosed in a high-risk myelodysplastic neoplasm patient with trisomy 8: a rare case report^☆^

**DOI:** 10.1016/j.abd.2024.11.003

**Published:** 2025-04-28

**Authors:** Renato Mendes Martins, Howard Lopes Ribeiro-Junior, Natália Feitosa Arrais Minete Mendonça, Fábio Rocha Fernandes Távora, Ronald Feitosa Pinheiro

**Affiliations:** a Center for Research and Drug Development, Universidade Federal do Ceará, Fortaleza, CE, Brazil; b Post-Graduate Program of Pathology, Universidade Federal do Ceará, Fortaleza, CE, Brazil; c Post-Graduate Program in Translational Medicine, Universidade Federal do Ceará, Fortaleza, CE, Brazil; d Post-Graduate Program in Medical Science, Universidade Federal do Ceará, Fortaleza, CE, Brazil

*Dear Editor,*

Pyoderma gangrenosum (PG) is a neutrophilic autoinflammatory dermatosis linked to dysregulated immune responses in genetically predisposed individuals. It involves elevated cytokines like TNF-α and interleukins (IL-1α, IL-17, IL-23), leading to cutaneous ulcers.^1^, ^2^ PG is often associated with systemic conditions such as rheumatoid arthritis, inflammatory bowel disease, and myelodysplastic neoplasms/syndrome (MDS), particularly in cases with trisomy of chromosome 8.^3^ This genetic abnormality may heighten inflammatory pathways, contributing to the severity of PG.^4^ PG frequently precedes MDS onset, suggesting its potential as a predictor of underlying hematologic disorders, although no cases linking high-risk MDS with trisomy 8 and PG were found.^5^, ^6^

Here, a 69-year-old female patient was admitted to a Brazilian tertiary hospital's emergency room with a one-month history of fatigue, lower limb pain, fever, and pancytopenia, showing a hemoglobin level of 7.4 g/dL, white blood cell count of 1830, and platelet count of 107,000 (Table 1). Initially, a bone marrow aspirate examination was conducted to explore the cause of the pancytopenia. The results revealed an 18% blast count (Table 1), indicating the possibility of MDS, specifically classified as the refractory anemia with excess blasts-2 (RAEB-2) subtype. A karyotype analysis was performed, which showed 47,XX,+8[11]/46,XX[1] (Fig. 1), classifying the patient as very high risk according to the Revised International Prognostic Scoring System (IPSS-R).

**Table 1:** Clinical and Laboratory Variables Before and After Progression to Acute Myeloid Leukemia (AML) in Myelodysplastic Neoplasm/Syndrome (MDS) Patients with Pyoderma Gangrenosum (PG).

Clinical Laboratory Variables	Before Progression	After Progression	Usual Range^a^
Hemoglobin (g/dL)	7.4	7	8 to <10
White blood cell count (×10^9^/L)	1.830	28.000	4.000–11.000 cells/µL
Neutrophils (×10^9^/L)	805	846	≥800 × 10^9^/L
Platelet (**/**mm³)	107.000	17.000	≥100.000
Blasts (%)	18%	37%	0‒30%

a Based on Revised International Prognostic Scoring System (IPSS-R).

**Fig. 1:**
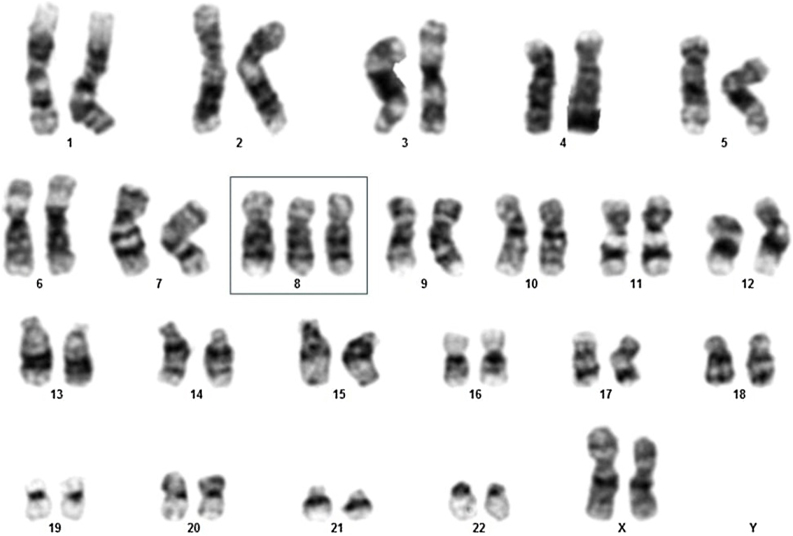
Karyotype analysis of elderly female myelodysplastic neoplasm/syndrome patient demonstrating 47,XX,+8[11]/46,XX[1].

She had a skin plaque on her left calf that evolved into a hemorrhagic blister, then an ulcer with irregular edges (Fig. 2). Initial treatments with piperacillin/tazobactam and vancomycin for a suspected infection did not lead to lesion improvement. A biopsy of the leg lesions revealed an infiltrate of mature neutrophils with epidermal ulceration consistent with PG (Fig. 3). The patient was prescribed dapsone 100 mg daily for 45 days to treat the lesion, with scheduled outpatient follow-ups. After completing the treatment, the lesions regressed, as shown in Fig. 4, and dapsone administration was discontinued.

**Fig. 2:**
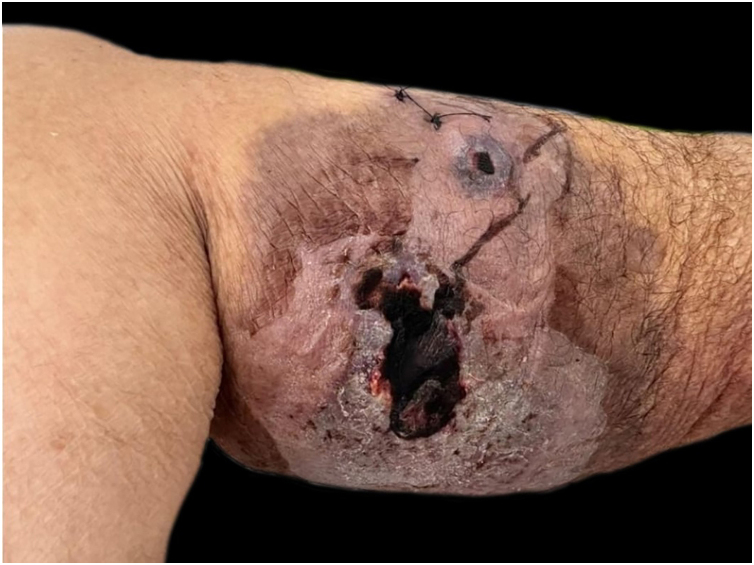
Lesion ulcerative with violaceous aspect on borders consistent with pyoderma gangrenosum diagnosis.

**Fig. 3:**
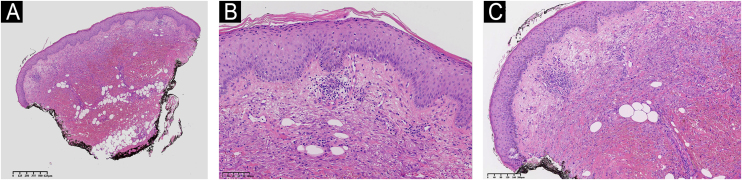
Histologic section of skin showing hyperkeratosis in the stratum corneum. The epidermis exhibits mild acanthosis. The dermis shows moderate inflammatory infiltrate characterized by lymphoplasmacytic leucocytes, with significant associated tissue hemorrhage. There is mild perivascular inflammation, without evidence of vasculitis. Absence of edema in the papillary dermis. (A) Low power of the histological section of skin demonstrating hyperkeratosis, mild inflammation and hemorrage. (B) Hyperkeratosis in the stratum corneum and mild acanthosis in the epidermis. (C) Moderate inflammatory infiltrate with a perivascular distribution and with tissue hemorrhage in the dermis. Hematoxylin & eosin staining.

**Fig. 4:**
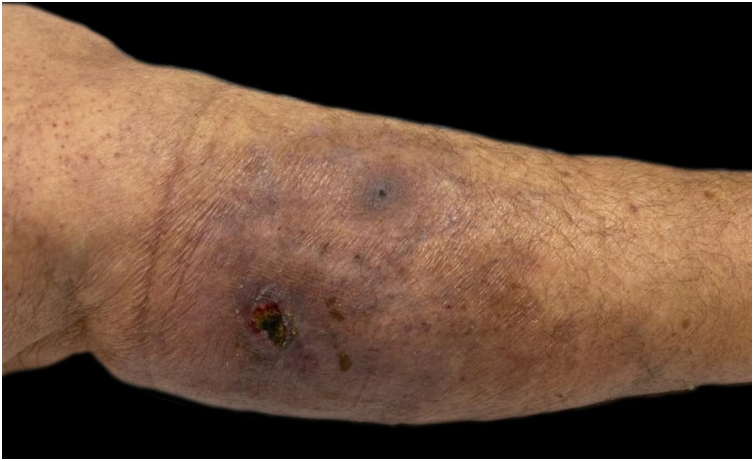
Pyoderma gangrenosum lesion regression after dapsone treatment.

However, after two weeks, the patient's hematological condition deteriorated, presenting with a hemoglobin level of 7 g/dL, white blood cell count of 28,000 (including 846 neutrophils and 26,000 blasts), and platelet count of 17,000 (Table 1). A subsequent myelogram revealed a blast percentage of 37% (Table 1), leading to a diagnosis of secondary acute myeloid leukemia (sAML) (Table 1). The patient was prescribed venetoclax100 mg and azacitidine 100 mg for 5 days with spaced by 21 days between applications for the treatment of sAML. Unfortunately, the patient died after 2 months of treatment due to a hemorrhagic stroke, which was attributed to severe thrombocytopenia. The study was carried out in accordance with CARE guidelines.

Our results demonstrated that the association of PG with hematologic disorders, particularly high-risk MDS and the presence of trisomy 8, suggests a deeper genetic and immunological connection that may influence both the presentation and treatment outcomes of affected patients. The first-line therapy for pyoderma gangrenosum is glucocorticoids, typically initiated at high doses (1–2 mg/kg) during the acute phase.^7^ However, this protocol was not followed due to the lack of feasible outpatient follow-up. Another possible therapeutic option is immunosuppression; however, neutropenia was considered a contraindication due to the high risk of infection, one of the leading causes of death in AML.^8^

Dapsone, beyond its use for bacterial infections as an inhibitor of bacterial folic acid synthesis, acts on myeloperoxidase-peroxide halide-mediated cytotoxicity ‒ a component of the neutrophil respiratory burst. It also inhibits the synthesis of chemotactic lipids and interferes with chemotaxis, reducing neutrophil migration to lesions. This medication was selected for treatment due to its wide availability and low cost if self-purchased, and its manageable side effects at low doses, such as areas of hyperpigmentation ‒ an acceptable trade-off compared to the risk of glucocorticoid-induced osteoporosis or high infection risk with immunosuppressants. Furthermore, the patient had no known drug interactions, and the prescriber had prior experience successfully using dapsone for PG, achieving lesion remission. The prescribing physician must tailor therapeutic approaches individually, considering each patient's needs, adherence potential, risks, and social circumstances to optimize recovery.

Additionally, similarly to our case, Haga and colleagues^9^ discuss mucocutaneous PG due to trisomy 8 neutrophilic infiltrates in an 87-year-old Japanese male patient with MDS, illustrating the complex pathophysiology that underlies this association and potentially guiding therapeutic decisions. Our case reinforces the clinical hypothesis that PG could be considered an external manifestation of the underlying hematologic disorder's complexity and severity. The aggressive nature of the skin lesions in PG, characterized by their rapid onset and resistance to conventional treatments, may parallel the progression of hematologic malignancies from a more indolent state to an aggressive, acute phase. Thus, PG might not only serve as a marker for the presence of an underlying hematologic disorder but could also indicate a turning point in the disease trajectory towards a more aggressive and less responsive state.

This hypothesis is supported by the notion that both PG and the progression of MDS to sAML involve dysregulated immune responses and inflammatory pathways. The genetic abnormalities associated with MDS, such as trisomy 8, could further exacerbate this dysregulation, leading to the manifestation of PG as a direct consequence of the underlying disease's progression. Moreover, the evolution of MDS to sAML, marked by an increase in blast cells and worsening cytopenias, might be mirrored in the skin by the worsening or uncontrolled progression of PG lesions.

In summary, we report for the first time a rapid response of PG lesion regression in a Brazilian patient with RAEB-2 MDS with trisomy 8 treated with dapsone. To date, data on high-risk MDS patients with trisomy 8 are rare in cohort studies and clinical follow-up. This case report highlights the association of the patient's immunological impairment with MDS, likely caused by the presence of trisomy 8, which may have triggered PG as a dermatological manifestation. Finally, this association underscores the intricate interplay between genetic abnormalities and immune dysregulation in the pathogenesis of hematological disorders with cutaneous manifestations, warranting further investigation and consideration in the clinical management strategies of MDS patients.

## Authors’ contributions

Renato Mendes Martins: The study concept and design; data collection or processing; analysis or interpretation; literature search; writing; final approval of the final version of the manuscript.

Howard Lopes Ribeiro Junior: The study concept and design; analysis or interpretation; literature search; writing; final approval of the final version of the manuscript.

Natália Feitosa Arrais Minete Mendonça: The study concept and design; data collection or processing; writing; final approval of the final version of the manuscript.

Fábio Rocha Fernandes Távora: Data Collection or processing; analysis or interpretation; writing; final approval of the final version of the manuscript.

Ronald Feitosa Pinheiro: The study concept and design; data collection or processing; analysis or interpretation; writing; final approval of the final version of the manuscript.

## Financial support

This research was funded by the Brazilian Ministry of Science and Technology supported by 10.13039/501100003593National Council for Scientific and Technological Development (CNPq) entitled: “UNIVERSAL CNPq/MCTI/FNDCT nº 18/2021 ‒ Faixa B ‒ Grupos Consolidados ‒ #422726/2021-4 ‒ *Análise da via STING de pacientes com Síndrome Mielodisplásica primária e de modelos de lesão de fita dupla de DNA secundária a quimioterápicos em pacientes oncológicos e camundongos* C57BL/6”. This research was funded by the Brazilian Ministry of Science and Technology supported by National Council for Scientific and Technological Development (CNPq) entitled: “CHAMADA CNPQ/MCTI/CT-BIOTEC nº 30/2022 ‒ *Linha 2: Novas tecnologias em Biotecnologia -* #440389/2022-4 *‒ Desenvolvimento de linhagens geneticamente modificadas por crispr/cas9 com perda de função do gene TP53: induzindo o fenótipo de instabilidade genômica da Neoplasia Mielodisplásica (SMD)*”. This research was funded by the *Instituto Nacional de Ciência e Tecnologia do Sangue (INCT do Sangue)* / #405918/2022-4). Howard Lopes Ribeiro Junior is recipient of a CNPq Research Productivity Scholarship ‒ Level 2 (Project: Chamada CNPq nº 09/2023 ‒ Bolsas de Produtividade em Pesquisa ‒ PQ #305659/2023-5). This research was funded by the *Fundação Cearense de Apoio ao Desenvolvimento Científico e Tecnológico* (FUNCAP) (UNI-0210-00007.01.00/23) entitled: “*Estabelecimento de um Escore de Risco Poligênico (PRS) para a Neoplasia Mielodisplásica no idoso: uma coorte Brasileira*”.

## Conflicts of interest

None declared.
